# Changing epidemiology of infections due to extended spectrum beta-lactamase producing bacteria

**DOI:** 10.1186/2047-2994-3-9

**Published:** 2014-03-25

**Authors:** Steven Z Kassakian, Leonard A Mermel

**Affiliations:** 1 Department of Medicine, Warren Alpert Medical School of Brown University, Providence, RI, USA; 2 Department of Epidemiology and Infection Control, Rhode Island Hospital, 593 Eddy Street, Providence RI 02903, USA; 3 Division of Infectious Diseases, Rhode Island Hospital, 593 Eddy Street, Providence, RI 02903, USA

**Keywords:** Extended-spectrum beta-lactamase, Urinary tract infection, Antimicrobial resistance, Community-acquired infections, Ciprofloxacin

## Abstract

**Background:**

Community-associated infections caused by extended-spectrum beta-lactamase (ESBL) producing bacteria are a growing concern.

**Methods:**

Retrospective cohort study of clinical infections due to ESBL-producing bacteria requiring admission from 2006-2011 at a tertiary care academic medical center in Providence, RI.

**Results:**

A total of 321 infections due to ESBL-producing bacteria occurred during the study period. Fifty-eight cases (18%) were community-acquired, 170 (53%) were healthcare–associated, and 93 (29%) were hospital-acquired. The incidence of ESBL infections per 10,000 discharges increased during the study period for both healthcare-associated infections, 1.9 per year (95% CI 1-2.8), and for community-acquired infections, 0.85 per year (95% CI 0.3-1.4) but the rate remained unchanged for hospital-acquired infections. For ESBL-producing *E. coli* isolates, resistance to both ciprofloxacin and trimethoprim-sulfamethoxazole was 95% and 65%, respectively but 94% of isolates were susceptible to nitrofurantoin.

**Conclusions:**

Community-acquired and healthcare-associated infections due to ESBL-producing bacteria are increasing in our community, particularly urinary tract infections due to ESBL-producing *E. coli*. Most isolates are resistant to oral antibiotics commonly used to treat urinary tract infections. Thus, our findings have important implications for outpatient management of such infections.

## Background

ESBL-producing bacteria cause infections in hospitalized patients [1-3], patients housed in long-term care facilities [4,5], and they are gaining a foothold in community settings [6,7]. Human fecal carriage with these microorganisms is increasing, as well as their ubiquity in non-human species [8]. The increasing prevalence of infections due to ESBL-producing bacteria creates a challenge regarding appropriate antimicrobial therapy, especially in the community setting where oral antibiotics are used.

Most ESBLs are found in *Escherichia coli* and *Klebsiella pneumoniae*, frequently harboring resistance to other classes of antibiotics [9,10]. The majority of infections caused by these pathogens are urinary tract infections with occasional secondary bloodstream infections. In general, the preferred antibiotic class for management of infections due to ESBL-producing bacteria are carbapenems [11]. The purpose of this study was to better understand the changing epidemiology of ESBL-producing bacteria.

## Methods

### Study population

This study was conducted at Rhode Island Hospital, a tertiary care hospital licensed for 719 beds in Providence, RI.

### Study design

This was an IRB-approved, retrospective cohort study of all adult patients hospitalized between January 2006 through December 2011 who had a positive clinical culture for an ESBL-producing microorganism.

### Microbiology

Clinical cultures were identified and tested for antimicrobial susceptibility utilizing the Vitek 2 System (bioMérieux, Inc. Durham, NC). The detection of ESBL in *E. coli* and *K. pneumoniae* was done as previously described [12]. The phenotype confirmatory test for ESBL production was performed with use of ceftazidime (30 μg) and cefotaxime (30 μg), with and without clavulanic acid, against the isolates. The discs were placed on pre-inoculated Mueller-Hinton agar and incubated at 37°C. A difference of ≥5 mm between the zone diameters of either of the cephalosporin disks and their respective cephalosporin/clavulanate disk was considered phenotypic confirmation of ESBL production.

### Demographic and clinical data

Cases were identified using infection control software (Theradoc, Hospira Inc. Lake Forest, IL). Cases were included if all three of the following were documented: an ESBL-producing microorganism was grown from a patient’s clinical specimen; the treating physician noted that the patient had an infection in the medical record; and the physician treated the patient with antibiotics. All charts were reviewed by one of the study authors (SK) to determine the infection acquisition type (i.e., community-acquired, healthcare-associated or hospital-acquired) using the Centers for Disease Control and Prevention definitions [13]. The antibiogram was obtained from the electronic medical record.

### Definitions

The site of infection was defined according to CDC definitions [14]. If a culture was obtained more than 48 hours after hospital admission, it was classified as hospital-acquired. If a culture was obtained within 48 hours after admission, it was classified as a healthcare-associated infection if a) within the prior 90 days the patient resided in a long-term care facility or, had an prior admission to an acute-care facility in our hospital system, or an outside hospital as mentioned in the admission note; b) or had undergone hemodialysis or received an intravenous medications or c) if they underwent an invasive procedure within the last 30 days prior to admission. Otherwise, a patient was considered to have a community-acquired infection.

### Statistical analysis

Age comparisons between the three groups were analyzed using a two-tailed t test. Sex differences among the three groups were analyzed using chi-square testing. Linear regression was performed to determine whether changes in the incidence of infection were statistically significant (SPSS, Chicago, IL). This analysis was repeated including only the first occurrence of an infection in a given patient. Differences in antibiotic resistance between acquisition groups were analyzed using either a chi-square test or a Fischer exact test when appropriate. Use of a two-tailed test of significance with a P-value <0.05 was employed to determine statistical significance.

## Results

**Figure 1:**
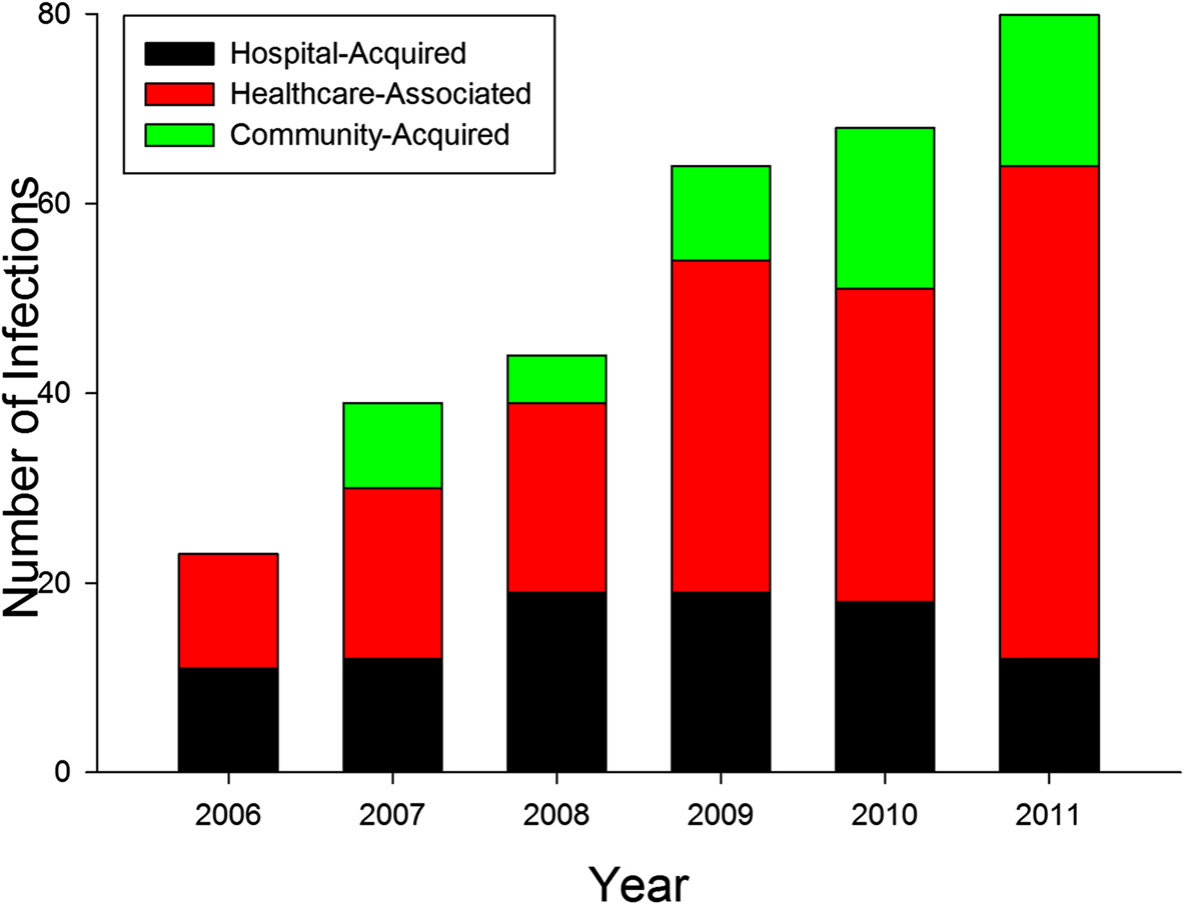
**Origin of infection due to ESBL-producing bacteria.** Incidence of infections due to ESBL-producing bacteria by classification of origin over the study period.

During the study period, there were 321 incident infections due to ESBL-producing bacteria. Twenty-six patients experienced more than one infection. One patient had two different ESBL-producing bacteria in the same clinical sample at one time. The number of infections due to these pathogens increased consistently from 23 infections in 2006 to 81 in 2011 (Figure 1). Overall, 58 cases (18%) were community-acquired, 170 (53%) healthcare–associated, and 93 (29%) hospital-acquired. The incidence of infection due to ESBL-producing bacteria per 10,000 discharges increased significantly during the study period for health-care associated infections, 1.9 per year (95% CI 1-2.8; p = .003) and for community-acquired infections, 0.85 per year (95% CI 0.3-1.4; p = .01). There was no significant change in the hospital-acquired infection group. When this analysis was repeated after removing 26 recurrent episodes of infections, none of the significant changes over time became non-significant (data not shown).

The mean age among the three groups was 69, 70 and 65 years in the community, healthcare and hospital-acquired infection groups, respectively. The difference in age between the healthcare-associated and the hospital-acquired infection groups was significant (p = 0.04). There were fewer males (26%) in the community-acquired group compared with healthcare-associated (42%) and hospital-acquired groups (41%; p = 0.1).

**Figure 2:**
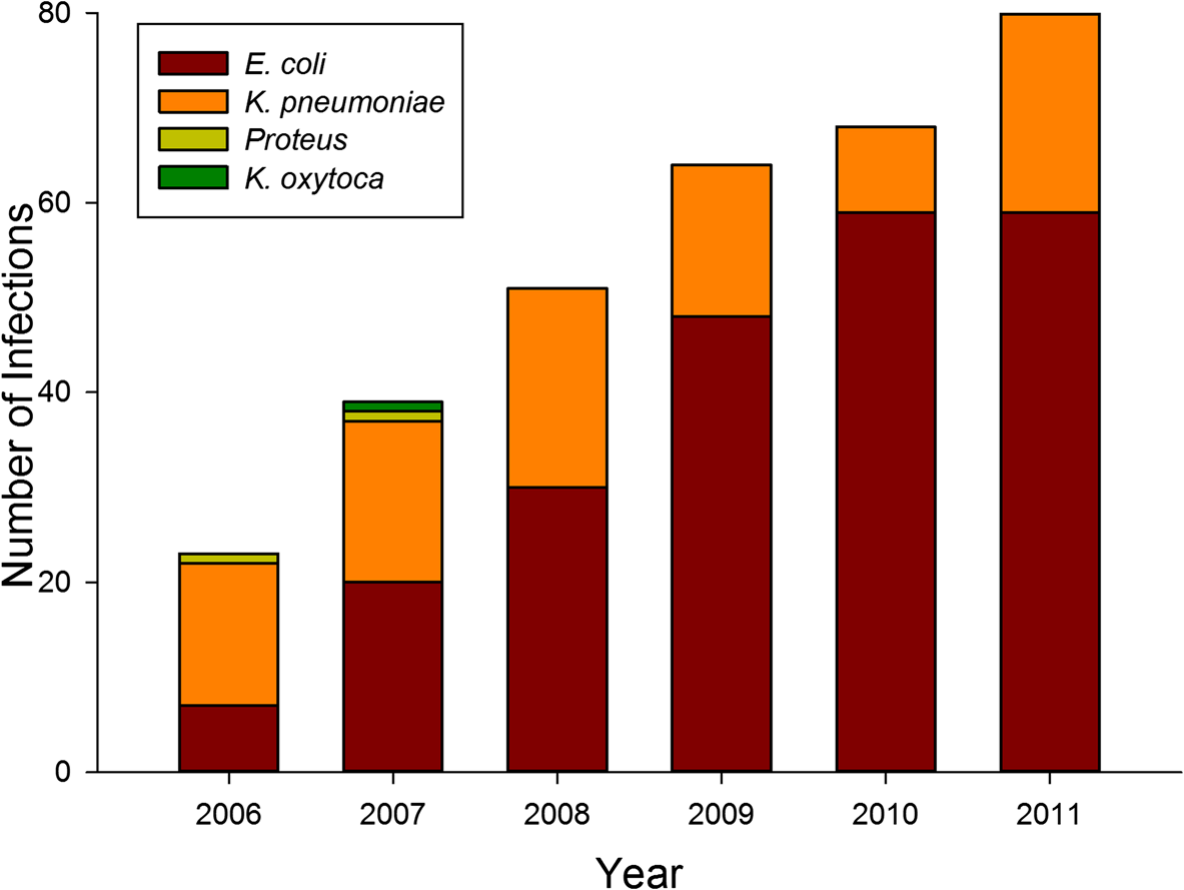
**ESBL-producing bacteria.** ESBL-producing bacteria identified during the study period.

Urinary tract infection predominated (80%), followed by bloodstream infection (10%), skin and soft-tissue infection (5%), pneumonia (3%) and intra-abdominal infection (2%). There was a marked shift in the predominant organism in all three acquisition types from *K. pneumoniae* to *E. coli* (Figure 2). For the entire study period, *E. coli* accounted for 78%, 66% and 65% of the community, healthcare-associated, and hospital-acquired groups, respectively.

Resistance to trimethoprim-sulfamethoxazole (TMP-SMZ) and ciprofloxacin was commonly observed among *E. coli* isolates (Table 1); however, 94% of *E. coli* isolates were susceptible to nitrofurantoin with no difference between acquisition groups (p = 0.8). In contrast, 76% of all *K. pneumoniae* isolates were resistant to nitrofurantoin.

**Table 1:** **Antimicrobial resistance of ESBL-producing *E. coli***

**Antimicrobial**	**Community acquired**		**Healthcare associated**		**Hospital acquired**		**Total**		**P value**
	**Bacteria**	**n(%) Resistant**	**Bacteria**	**n(%) Resistant**	**Bacteria**	**n(%) Resistant**	**Bacteria**	**n(%) Resistant**	
TMP-SMZ*	51	32 (69)	111	75 (68)	57	45 (79)	219	152 (69)	0.2
Ciprofloxacin	52	48 (92)	111	109 (98)	57	53 (92)	220	210 (95)	0.1
Nitrofurantoin	50	3 (6)	85	7 (8)	43	1 (2)	178	11 (6)	0.5

Legend: *Trimethoprim-sulfamethoxazole.

## Discussion

The number of infections due to ESBL-producing bacteria are increasing, especially community or non-hospital healthcare-associated infections as demonstrated by others [15,16]. Although the focus of infection control measures has been on transmission of such pathogens within hospitals, many of the infections due to ESBL-producing bacteria appear to arise outside of the acute-care setting where there is limited infection control resources [17].

We found high levels of resistance to TMP-SMZ and ciprofloxacin in all acquisition types. *E. coli* resistance to TMP-SMZ was similar to other studies [18]; however, ciprofloxacin resistance among *E. coli* in our study (95%) is higher than found in other reports [10,15,16,19-21]. The level of ciprofloxacin resistance we documented in our community-acquired ESBL-producing *E. coli* (92%) is also higher than the level of resistance among all *E. coli* isolates tested at our hospital in 2011 (31%) [22]. While it is known that many ESBL-producing bacteria harbor additional resistance genes, it remains unclear why our isolates had such high levels of ciprofloxacin resistance. One possibility is that the CTX-M was the predominant gene regulating beta-lactamase production in most of our isolates. While we did not investigate the molecular mechanisms of resistance in our study, we wonder whether our isolates which showed the persistently increasing predominance of *E. coli* may indeed be the advance of CTX-M in our region, as has been found in rectal colonizers in other patients admitted to our hospital (L.A. Mermel, unpublished data, February 2013). Of note, a US multi-centered study documented 99% ciprofloxacin resistance among CTX-M-containing *E. coli*[23] and a recent US multi-centered study of community-acquired *E. coli* ESBL infections found the majority contained CTX-M genes [15]. This finding raises concern for treatment failure in the community setting given the levels of resistance to antimicrobial agents commonly used for cystitis, namely TMP-SMZ and ciprofloxacin. We found a low level of nitrofurantoin resistance in our *E. coli* isolates. As such, patients in our study with community-acquired infections may have been outpatient treatment failures owing to initial empiric therapy with either ciprofloxacin or TMP-SMZ, prompting hospital admission. While we did not have susceptibility data to fosfomycin, a recent study found no resistance among community-acquired ESBL-producing *E. coli*[16]. Thus, it seems prudent to consider the use of nitrofurantoin or fosfomycin as empiric therapy for acute uncomplicated cystitis in those at risk for, or with a history of infection or colonization with an ESBL-producing *E. coli*.

What accounts for the increasing numbers of ESBL-producing *E. coli* and the increase in community-acquired infections? Traditionally, *K. pneumoniae* made up the majority of infections due to ESBL-producing pathogens and the majority of those occurred in hospital settings. However, 88% of our community-acquired isolates of ESBL-producing bacteria in 2011 were *E. coli*, a marked shift from four years earlier when it was 55%. The presence of ESBL-producing *E. coli* as commensal flora in healthy livestock has been documented throughout Europe and Asia with as many as 40% of poultry populations colonized with these bacteria and their presence has been identified in retail meats [8,24,25]. Additionally, several studies have found ESBL-producing *E. coli* in the fecal flora of healthy companion animals, namely cats and dogs, and recently such isolates have been detected in US animals, where the predominant strain was CTX-M [8,26]. Beyond domesticated animals, ESBL-producing *E. coli* have been found in wildlife in several continents and many such isolates have been shown to be genotypically-related to human isolates [27].

Regarding limitations in our study, it is possible that patients were misclassified as community-acquired given the lack of available documentation in the medical record. Given the lack of admission screening, it is possible that some cases deemed hospital-acquired infections were community-acquired. Our community-acquired cohort is likely a biased subset as they required hospital admission, thus indicating that they likely were more ill than those whom developed such infections and remained in the community. Additionally, our antibiotic resistance patterns for the community-acquired and healthcare-associated organisms likely present a biased sample as only those patients ill enough to require admission are represented. Use of the Vitek 2 system alone for identification of ESBL-producing *Enterobacteriacae* is insufficient [28]. As such, our laboratory used confirmatory testing as noted in the Methods section. Lastly, our laboratory uses CLSI breakpoints for susceptibilty testing. The CLSI has updated recommendations for interpretation of antibiotic susceptibility testing results in the 2010 and 2011 CLSI guidelines, in part adopting European Committee for Antimicrobial Susceptibility Testing (EUCAST) strategies. The CLSI now recommends higher zone diameter susceptibility breakpoints for 3^rd^ generation cephalosporins and carbapenems; fluoroquinolone breakpoints were unchanged. Thus, our data must be interpreted in context, based on previously used CLSI breakpoints rather than revised CLSI breakpoints or those recommended by EUCAST [29].

## Conclusion

In summary, our study noted the emergence of community-acquired infections due to ESBL-producing bacteria, a marked increase in healthcare-associated infections, as well *E. coli* becoming the predominant pathogen in all three acquisition groups. We found high levels of TMP-SMZ and ciprofloxacin resistance. This has implications regarding empiric therapy for urinary tract infections since these frequently utilized antibiotics in the outpatient setting are ineffective for such pathogens. Another important finding is the susceptibility of ESBL-producing *E. coli* to nitrofurantoin. Further elucidation of underlying genetic makeup of ESBL-producing pathogens will assist in better understanding the epidemiology of these emerging infections.

## Abbreviations

ESBL: Extended-spectrum beta-lactamase; TMP-SMZ: Trimethoprim-sulfamethoxazole.

## Competing interests

The authors declare that they have no competing interests.

## Authors’ contributions

SZK and LAM are both fully responsible for the entirety of the study from conception through critical revisions of the manuscript. Both SZK and LAM had full access to all the data in the study and take responsibility for the integrity of the data and the accuracy of the data analysis. Both authors approved the final manuscript.
